# An innovative leadership development initiative to support building everyday resilience in health systems

**DOI:** 10.1093/heapol/czab056

**Published:** 2021-05-18

**Authors:** Jacinta Nzinga, Mwanamvua Boga, Nancy Kagwanja, Dennis Waithaka, Edwine Barasa, Benjamin Tsofa, Lucy Gilson, Sassy Molyneux

**Affiliations:** Health Services and Research Group, Kenya Medical Research Institute/Wellcome Trust Research Programme, PO Box 43640, Nairobi 00100, Kenya; Health Services and Research Group, Kenya Medical Research Institute/Wellcome Trust Research Programme, PO Box 43640, Nairobi 00100, Kenya; Health Services and Research Group, Kenya Medical Research Institute/Wellcome Trust Research Programme, PO Box 43640, Nairobi 00100, Kenya; Health Services and Research Group, Kenya Medical Research Institute/Wellcome Trust Research Programme, PO Box 43640, Nairobi 00100, Kenya; Health Services and Research Group, Kenya Medical Research Institute/Wellcome Trust Research Programme, PO Box 43640, Nairobi 00100, Kenya; Nuffield Department of Medicine & Department of Paediatrics, University of Oxford, Henry Wellcome Building for Molecular Physiology, Old Road Campus, Headington, Oxford OX3 7BN, UK; Health Services and Research Group, Kenya Medical Research Institute/Wellcome Trust Research Programme, PO Box 43640, Nairobi 00100, Kenya; School of Public Health and Family Medicine, Faculty of Health Sciences, University of Cape Town, Observatory, 7925 Cape Town, South Africa; Department of Global Health and Development, London School of Hygiene & Tropical Medicine, Keppel Street, London WC1E 7HT, UK; Health Services and Research Group, Kenya Medical Research Institute/Wellcome Trust Research Programme, PO Box 43640, Nairobi 00100, Kenya; Nuffield Department of Medicine & Department of Paediatrics, University of Oxford, Henry Wellcome Building for Molecular Physiology, Old Road Campus, Headington, Oxford OX3 7BN, UK

**Keywords:** Health systems, health system leadership and governance, leadership capacities, health managers

## Abstract

Effective management and leadership are essential for everyday health system resilience, but actors charged with these roles are often underprepared and undersupported to perform them. Particular challenges have been observed in interpersonal and relational aspects of health managers’ work, including communication skills, emotional competence and supportive oversight. Within the Resilient and Responsive Health Systems (RESYST) consortium in Kenya, we worked with two county health and hospital management teams to adapt a package of leadership development interventions aimed at building these skills. This article provides insights into: (1) the content and co-development of a participatory intervention combining two core elements: a complex health system taught course, and an adapted communications and emotional competence process training; and (2) the findings from a formative evaluation of this intervention which included observations of the training, individual interviews with participating managers and discussions in regular meetings with managers. Following the training, managers reported greater recognition of the importance of health system software (values, belief systems and relationships), and improved self-awareness and team communication. Managers appeared to build valued skills in active listening, giving constructive feedback, ‘stepping back’ from automatic reactions to challenging emotional situations and taking responsibility to communicate with emotional competence. The training also created spaces for managers to share experiences, reflect upon and nurture social competences. We draw on our findings and the literature to propose a theory of change regarding the potential of our leadership development intervention to nurture everyday health system resilience through strengthening cognitive, behavioural and contextual capacities. We recommend further development and evaluation of novel approaches such as those shared in this article to support leadership development and management in complex, hierarchical systems.

**Key messages**

- Health managers are key actors in all health systems, and therefore central to system resilience, but they are often unprepared and unsupported in leading and managing with particular inadequacies in support for interpersonal relationship elements of their work (communication skills, management of emotions, supportive oversight, etc.).
- We share our experiences from Kenya of designing and implementing a participatory course that provided contextually embedded soft skills and may provide lessons for those seeking to understand and develop leadership in other LMIC healthcare settings, wherein such research remains underdeveloped.
- The relationship between researchers and managers was an important aspect of the intervention, it encouraged managers to start initiating their own processes for change, such as peer support and mentoring and speaking out.
- Embedded, participatory, reflective and experienced learning approaches stimulates motivation to learn, ownership of process and empowerment to use skills in practice.

## Introduction

To strengthen the capacity of the health system to absorb, adapt and transform in the face of challenges, and thereby be ‘resilient’, health system actors must embrace the system’s complex and adaptive nature (Gilson *et al.*, 2017). Everyday health system resilience (Barasa *et al.*, 2017a) leverages on the interaction of the system’s hardware (technology, infrastructure, funding and human resource) and software elements (skills, knowledge, decision-making processes, values, norms, relationships and communication practices) (Aragón, 2010; Elloker *et al.*, 2012). Prior research has shown that organisational software is at least as important as organizational hardware in nurturing health system resilience (Gilson *et al.*, 2020; Kagwanja *et al.*, 2020). Specifically, leadership practices that recognize complexity and facilitate inclusive decision-making and forging of connections among system agents have been shown to improve system resilience (Gilson *et al.*, 2020).

In low- and middle-income countries (LMICs), district health systems play a critical role in the delivery of primary healthcare (PHC), basic services and training of healthcare professionals. Many managers operating within district systems are ‘mid-level’ managers, playing and important and challenging boundary-spanning role between facility staff and managers’ higher-up health system hierarchies. However, managers at this level of the system are often unprepared and unsupported in leadership and management (Egger and Ollier, 2007; Nzinga *et al.*, 2018). Some may have received *ad hoc* leadership and managerial training, but this training tends to focus on technical aspects of leading and managing. Relational competencies are generally overlooked and training is rarely carefully tailored to the specific context within which health managers work (MSH, U., Mom Services And Moph Sanitation, 2008; Kebede *et al.*, 2012). There is therefore need to develop innovative, context-specific ways of preparing and supporting health managers to lead in difficult and complex environments (Reich *et al.*, 2016; Gilson and Agyepong, 2018). In developing context-specific health leadership development interventions, the value of on-site and on-the-job training for health managers using innovative approaches such as reflective learning and systems thinking principles has been recognized (Edmonstone, 2015; Doherty *et al.*, 2018). These approaches acknowledge the importance of novel, contextualized forms of leading such as distributed and participatory leadership and aim to build the relational aspects of health systems (Fulop and Mark, 2013; Cleary *et al.*, 2018; Nzinga *et al.*, 2018).

The authors of this article are health policy and systems researchers involved in long-term collaborations with health system actors across Kenya, including embedded governance research in one Kenyan county (Molyneux *et al.*, 2016). Drawing on the literature and our experience and relationships, we co-developed with managerial colleagues a package of interventions to support them in performing their leadership and managerial roles. These interventions included:

- Locally adapting a taught course on complex health systems covering conceptual skills and practical tools for understanding and navigating health system complexity;
- Reorientating a course on communication skills and emotional competence for health workers to suit the needs of mid-level health managers; and
- Reflecting regularly with managers on the everyday challenges of their jobs, coping strategies and key areas of support needed.

In this article, we describe the leadership development training co-developed by us with the managers, and share findings from a formative evaluation of the training. We provide insights on how the initiatives were perceived by health managers and how their learning started influencing their everyday leadership practice. As reported elsewhere (Diahls/Resyst, 2020), we were able to draw on our broader embedded governance research activities, including regular reflection with managers, to understand the potential of the leadership interventions to influence managers’ relationships with subordinates, colleagues and supervisors.

## Study setting and methods

The leadership training we co-developed with managers was part of a broader and long-term programme of embedded governance research (Gilson *et al.*, 2017; Diahls/Resyst, 2020). The research approach centred on one county in Kenya, Kilifi, which became a ‘learning site’, comprising cycles of research and regular, reflective engagements between researchers and decision makers around decision-making challenges and research findings. Learning site engagements made use of a common approach that builds on participatory action-learning methods, including collective enquiry (where researchers and managers took part in regular cycles of planning, implementation and reflection around the activities), multiple methods (document reviews, interviews and observations of meetings and interactions) and reflective practice (Barasa *et al.*, 2020). Regarding reflective practice, the research team held regular meetings among themselves and with managers to provide space for conversation and critical reflection. Definitions of reflective practice emphasize purposeful critical analysis of knowledge and experience, in order to achieve deeper meaning and understanding (Loughran, 2002). Our past work guided the identification of mid-level managers (sub-county managers at meso-level) as key staff to focus on in examining and strengthening health system leadership, given their boundary spanning role between facility in-charges (micro level) and county health (macro-level) managers. Facility managers are also important given the complex and diverse roles they perform in difficult, sometimes relatively isolated environments, relying on the support of mid-level managers, but with relatively little formal preparation and training (Nzinga *et al.*, 2013; Nyikuri *et al.*, 2015; 2017). From our early engagements with health managers, we recognized the knowledge and experience managers had from their exposure to the system and their interest to be future leaders. However, we were also aware of the challenges they faced in managing the dynamic complexities at the various levels of the health system for which they were responsible.

We first describe the leadership initiatives we developed, how these were delivered and then provide an overview of the evaluation approach we adopted in understanding their influence.

## Developing and implementing a multifaceted leadership development programme

We adapted two specific courses to support leadership development among mid-level managers and facility managers.

### Introduction to Complex Health Systems

As Kenyan based researchers, we had the opportunity to participate in a short course run in South Africa entitled Introduction to Complex Health Systems. This open source course was developed in 2015 by Collaboration for Health Policy & Systems Analysis in Africa (CHEPSAA), a learning and teaching network for teachers, researchers, students and policy networks aimed at building the field of Health Policy and Systems Analysis (https://www.hpsa-africa.org/). We saw the potential value of the course for our manager colleagues, and therefore worked with them to adapt the course for the Kenyan setting. We named it ‘Understanding Dynamic Health Systems’, and ran it in partnership with a local University.

For this course, we made three main adaptations. First, we revised the leadership and management module to include theory behind emotional and social intelligence (Kerr *et al.*, 2006; Crowne *et al.*, 2017) and to provide practical case studies of delegating and role modelling. The revised course included engaging with managers in reflecting upon and discussing why and how to practically develop relational leadership abilities through improved communication skills and emotional competency (thus providing an introduction and link to the second course, described in the following). Second, we added group work on unpacking a health system challenge and dedicated an entire day to teams working through identifying strategic priorities to address a health system challenge they were facing and developing work plans to address those challenges. For the latter, we drew on the Challenge Model (https://www.msh.org/resources/the-challenge-model) which provides a logical process of identifying and achieving desired measurable results. This model has been used in leadership programmes locally to inspire teams with confidence (Lemay and Ellis, 2008a; Chelagat *et al.*, 2021). Third, we included case studies of research from Kenya and where possible from the county itself to strengthen contextual relevance (Barasa *et al.*, 2017b; Waweru *et al.*, 2013; Tsofa *et al.*, 2017). In adapting the course, we were cognizant of balancing teaching new cognitive understandings and technical skills (i.e. health system frameworks, systems thinking, understanding health system complexity, how to perform stakeholder analysis) and building critical thinking skills (i.e. diagnosing and framing problems differently, understanding organisational contexts and using relational skills in managing change). Important new elements included an introduction to systems thinking and health system complexity (Ortiz Aragón, 2010; De Savigny and Adam, 2009; Sheikh *et al.*, 2011), including the interaction of hardware and software elements within any organizational system (Table 1).

**Table 1.** Activities and concepts taught during the ‘Understanding Dynamic Health Systems’ course

Concepts taught/used	Activities involved
**Definition of a health system** and health system boundaries; **Frameworks for analysing health systems**; **Centrality of people** within the health system and the conceptualization of the health systems as comprised of ‘hardware’ and ‘software’, which are both critical for optimal functioning of the health system **Complexity** in health systems and the importance of agents and their mindsets, interests and power; Stakeholder analysis Principles **of systems thinking** and whole systems change and how managers can apply this in everyday leadership and management **Leading change** in health systems **Leadership styles** for different situations, engaging with everyday politics and building relational and interpersonal skills for managing people and change Introduction to and working with the **Challenge model** to identify and solve a local system problem	Integration of **local case studies** (drawn from past work) into CHEPSAA course as relatable examples Additional components: Participants identify a **health system problem** they face and apply skills taught in the course to the problem. **Work-based reflection and team activity** to work through an agreed and prioritized problem to achieve a desirable measurable result **Using the Challenge model**, they work through the identified problem back in their workplace and present results of agreed on measurable result during subsequent reflective meetings Approach: Involved class-based lectures, group work and audio-visual teaching materials

The adapted course was run in June 2017, with 30 participants from three county health management teams in the region attending the 5-d training.

### Communication skills and emotional competence course

Building on the first course and specifically aimed at developing managers’ abilities to nurture positive relationships and values within their organisational contexts, we developed a follow-on course for the same groups of health managers. For this more in-depth training process we targeted managers from only one of the three county health management teams involved in the previous course.

To plan the training process we drew heavily from an existing international participatory training course on communication skills and emotional competence developed and implemented in collaboration with physicians and nurses in nine countries over a period of 14 yr; over 300 health professionals having been trained using this approach [https://connect.tghn.org/resources-and-training/training/icare-haaland-model/]. Since then there have been various adaptations to suit different audiences. We worked with managers to include new modules on supportive supervision, facilitative feedback, developing trust and managing relationships.

The course included a special emphasis on using reflective practice to support self-awareness and building personal and professional relationships using participatory principles (Table 2). Course facilitators—two senior Kenyan nurses and trainers—had been trainers on the original course for frontline health workers and were experienced in creating a supportive learning environment wherein participants felt safe to share experiences and real-time problems. With our manager colleagues and participants, we held a one day deliberative meeting to adapt the course content and plan the training processes/phases and timing in such a way as to be acceptable and realistic to both trainers and managers (Table 2). Ten participants began the training process, but two were unable to complete it.

**Table 2.** Training phases for the communication skills and emotional competence course

Phase	Activities/content covered
Phase 1: July—Sept 2017	One-day **planning meeting** with managers to understand challenges and learning needs **Baseline** questionnaire to asses individual communication strengths and challenges **Self-observation ‘in action**’ and reflection to discover communication behaviours and effects on others: using guided weekly tasks on a set of specific aspects of communication and emotional management. Aim to strengthen participants’ self- awareness and start a change process. **Monthly reflective meetings** where there was collective learning based on individual’s own reflections in the workplace (to build coherence on what skills are needed to address needs in a & b above) **Reflective assignments pack 1: Basic communication skills** **Week 1: Listening:** How well do you listen to your colleagues, juniors, others and effects? **Week 2: Asking questions:** How do you discuss, and ask questions when dealing with colleagues/juniors? **Week 3: Discussion habits:** When discussing with Juniors/colleagues, do you hinder or inspire good communication?
	**Reflective assignments Pack 2: Handling emotions** Set of tasks aimed at building emotional competence skills. Participants observed for 1 mo on: Recognition of own and other people’s (juniors & colleagues) emotions and effects on communication. Reflect on strategies they use to manage their emotions
Phase 2—Sept 2017 Skills workshop	A short **participatory skill-building course of 3 d** using experiential learning methods tailored to manager needs linking participants observation and reflection to theory (the original course has a 1-wk skills building, but we adapted it for this group given other routines and responsibilities) **Content covered:** Basic communication skills Managing emotions with awareness Managing conflict to maintain dignity and respect Supportive supervision Using power with awareness
Phase 3—Sept 2017 to March 2018	**Continued self-observation and reflection** after the skills building course on the application of new ideas and skills in practice, using specific reflective assignments to deepen and confirm learning **Reflective assignments** Pack 3: Strengthening communication with colleagues Pack 4: Communication with supervisors, taking care of safety and emotions.
Phase 4—March 2018 Skills workshop	**A two-and-half days follow-up workshop (5 mo later)** for collective learning and reflection, in which learning was summarized and anchored to daily challenges and best practices to strengthen confidence and empowerment **Content covered** Review of communication strategies with colleagues What makes people change attitudes and behaviour? And why don’t my juniors change? Why and how to recognize and manage and prevent burnout Recognizing bullies at the workplace: Taking action to confront and prevent bullying Many phases of anger: Recognize, acknowledge and handle with respect. Dealing constructively with conflict: From confronting—to stepping back and dialogue.

## Evaluation approach for the two courses

We designed a formative evaluation aimed at contributing to further development of leadership initiatives rather than addressing the summative question of whether the training was effective or how can it be modified in ways that increases potential for effectiveness. We tracked how these different initiatives unfolded over a period of 18 mo (Sept 2017–Dec 2018), drawing on data collected from three approaches: activities built into the courses themselves, specifically developed interviews, and broader learning site engagements.

**Figure 1.**
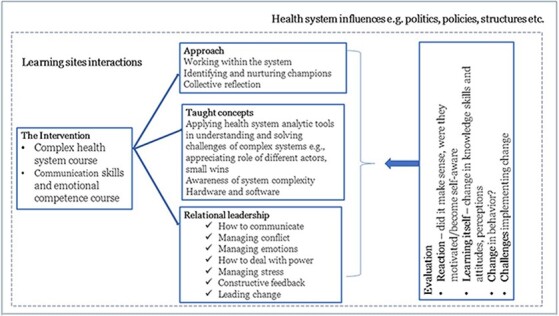
A conceptualisation of how we approached the evaluation of the multifaceted leadership development initiatives

To guide our evaluation, we used the Kirkpatrick model accepted and used extensively for the last 30 yr to assess training activities (Alyahya and Norsiah, 2013; La Duke, 2017). Using Kirkpatrick model’s four levels of evaluation, we explored: (1) participant’s reaction to the training style (e.g. satisfaction with the training); (2) participants’ understanding of the training (e.g. increase in knowledge, skills or experience), (3) whether they are utilizing what they learned at work (e.g. change in behaviours) and (4) whether the material had a positive impact on their jobs (Kirkpatrick, 1994). We considered the potential of reflective practice and experiential learning to support the spread of leadership capacities among health managers at facility, sub-county and county levels and the implications on the practice of everyday health system resilience. Figure 1 summarizes our evaluation approach.

The focus of our evaluation was on the communication skills and emotional competency course elements because of the novelty of the approach and its’ focus on elements anticipated to strengthen relational leadership, health system software and—ultimately—everyday health system resilience.

### Activities built into the courses themselves

Notes were made during planning meetings between managers and researchers on the content and delivery of the trainings. Pre- and post-activity evaluations were built into the communication and emotional competence course, including: a self-administered baseline questionnaire; a workshop evaluation drawing from anonymized diaries/transcriptions; and requests for participants to share ‘most significant change stories’ from reflective assignments (Dart and Davies, 2003). These data were supplemented with tracking verbal and non-verbal reactions to different course sessions and participant interactions during training workshops.

### Specifically developed interviews

We conducted in-depth interviews with all health managers who had attended both the complex health systems and the entire communications skills training (*n* = 8). They included subcounty health managers (4), hospital managers (3) and peripheral facility managers (2). Interviews focused on managers’ experiences of using concepts and skills gained during the trainings in their routine work and what opportunities and challenges they faced in bringing about change in their jobs.

### Broader learning site engagements

Our evaluation was strengthened through ongoing learning site interactions with managers; both those who had and had not participated in the training. These interactions allowed us to track how initiatives and agreements from courses were being implemented and prioritized, if and how they were influencing health managers’ leadership practices and organisational relationships, as well as broader influences and concerns. They also allowed us to draw upon the tacit knowledge of the managers and their experience to strengthen the ongoing adaptation of the second course with the aim of strengthening its’ relevance and impact.

Our overall evaluation approach was focused on how the learning activities allowed the managers to reframe daily challenges, use relevant tools to understand complexity in attempting to solve these challenges and strategically use relationships and interactions with other agents in the system. Over time through reflective meetings, feedback meetings on research findings and informal discussions, we were able to consider the influence of the trainings on behaviour and attitude change at work and how learning was spread.

The specific data drawn upon to analyse the impact of the leadership training are summarized in Table 3.

**Table 3.** Nature and source of data

Types of data	Details
Workshop planning notes	Notes taken from a 1-d planning meeting
Baseline questionnaire	10 questionnaires on individual assessment on communication and emotional competence strengths and challenges
Workshop observation notes	Notes taken from the 3-d skills training workshop on communication skills and emotional competence
Personal reflections of the health managers recorded in the diaries from the reflective assignments in phases 1 and 3, and one on one discussions with managers	Most significant stories of change from 10 course participants
Evaluation questionnaires for the ‘Understanding complex health systems course’ Tracking what modules were most valuable to individuals Modules of most relevance to current work Perception of facilitation style and effectiveness in communicating key learning Exploring what was generally found to be most enjoyable during the course	10 questionnaires collected from managers at different levels of the health system
Workshop evaluation self-administered questionnaires Tracking change in leadership and management practices	10 questionnaires collected from managers at different levels of the health system
Communicating with others	
Managing own emotions and that of others	
Overall influence on leadership styles	
Post-workshop evaluation	
In-depth interviews	Nine interviews with health facility managers with sub-county health facility managers
Notes from reflective discussions between research team and managers	Quarterly meetings over the year lasting approximately 1.5 to 2 h each

## Data analysis

Our overall analysis used a thematic approach while ensuring integration of the data sources summarized in Table 3 above (Cronin *et al.*, 2008). We started by inductively coding each of the data sources, identifying a thread within a data source and then following it to the next data source. Guided by the Kirkpatrick model, we thematically categorized data into themes by relating each identified thread to the study objectives and relevant literature. For interpretation, findings were discussed among the research study team, other members of the learning sites in Kenya and South Africa, and then feeding back to health mangers for member checking during reflective meetings. Final themes synthesized from interrogation of all data sources were iteratively refined to determine their contribution to the research question, guided by Kirkpatrick’s model. This process resulted in four broad thematic areas presented in the results section. In the discussion we return to the analysis linked to level 4 of the framework; reflections on how the training started influencing personal and team capacities that contribute to strengthening everyday resilience of the system.

Data collected about the influence of the communication course on managers was, in part, undertaken by two team members centrally responsible for design, implementation and evaluation of the course. We were reflexive about our positionality during analysis and interpretation of the data—using a consultative process of discussion with other research team members who were not part of the implementing group in coding transcripts and interpreting findings. Furthermore, presentation and discussion of emerging thematic areas with our colleagues from the South African learning sites allowed additional researcher reflection and minimized biased results.

## Results

As noted above, 30 mid-level managers participated in the complex health systems course from across three county health management teams. Managers from one county health management team were invited to either participate themselves or to nominate a suitable team member to participate in the communication skills and emotional competence course. A total of 10 managers participated in the latter course. Our results centre on the inputs of the eight managers who underwent the full training. Of the eight, three were sub-county health managers with 3–7 yr working experience and five were senior facility-based managers (four with clinical backgrounds and one a hospital administrator). Three of the eight had had management training of some form before this course.

Here we summarize managers’ perceptions and experiences of the training under four thematic areas: managing self, experiencesof implementing the course, skills and competencies acquired and application and spread of the course. In the discussion we draw on these findings and the literature to consider the implications of the training approach for strengthening everyday health system resilience.

### Managing self

#### Changes in managing emotions

During the communication and emotional management training managers reported that the most relatable and practical soft skill learned was ‘being aware’. They reported being more aware of themselves and of the situation, and of being able to ‘step back’ to assess situations before engaging. Moreover, they reported improved communication as a result of listening without interruption using taught skills such as ‘listening more - talking less’, ‘ensuring everyone voices their opinion’ and being conscious ‘not to cause harm to oneself or to colleagues’.

*Sometimes I would view my juniors as people who deserve no attention, but now I respect both my juniors and supervisors recognizing that even my supervisors have their own weakness(es). I am now:*

- *Self-aware about emotions and able to scan environment before responding*
- *Able to appropriately delegate task and allow team members to enjoy the sense of self-achievement*

These changes were described as being facilitated by learning to unlearn old behaviours and attitudes, being more aware, thinking more about behavioural consequences and building confidence in challenging the status quo. Changes were presented as personal change in disposition, change in intrinsic drive and using personal innovation and resources, e.g. leveraging on relationships, as described in the following.

#### Strengthened confidence and improved agency in work

Most health managers reported being more confident at tackling work issues, emphasizing that while they did not always find solutions for problems, they felt better prepared to start doing something to change situations. Examples included courage to report harmful/dishonest practices, courage to vent out/share emotions and personal initiatives in finding solutions. However, all managers recounted that these changes required significant unlearning of old habits.

*… for me the purpose of that training really refined my thinking as a manager because, you know you’re a manager you’re told, ‘Okay, supervise people, do this, do this, routine clock-work things and you’re good to go,’. But now when you take such training, your sense of reasoning, for me it actually widened my horizon and thinking… and emotionally I have learnt to not …. Before the training I could even say let me walk out of this it’s too much* (Hospital manager, reflective meetings)

#### Developing respect and showing empathy

Managers revealed a growing ability to identify with the collective emotional state of others as exemplified during a nationwide nurses’ strike^1^ period, wherein several nurses continued to work for the wellbeing of patients. A nurse manager recounted how when approached by angry nurses regarding understaffing from the strike, she showed empathy and hope:

*Regarding my colleagues, I imagine if it’s me with that workload. Would I have been able to complete the task on time and in the required standard? Empathy helps me deal with my staff well. I recognize what they are going through and what they are able to do. They feel mama yuko na sisi [The matron is with us]* (Interview, sub-county nurse manager)

Another manager reportedly appealed to the altruism of willing nurses to continue coming to work, while protecting nurses who continued to work from violence and threats of those who were on strike.

There were reports that constructive feedback only worked when intentions were explicitly spelt out and when a personalized and individualized approach was used. When delivered correctly it was perceived as empowering and motivating to recipients.

*I give better feedback if I am aware of what I want to achieve from that communication. When I want to achieve a certain objective, for example managing a sick patient, I say specifically what was done right and by whom and this helps cement the confidence of the performer as well as encourage imitation of the good work by others on the team.* (Interview, nurse-charge)

Linked to unlearning unhelpful habits were managers’ shared reflections on how to practice and sustain positive social competences by acknowledging that changing behaviour is, ‘*a process and a journey, not a destination*’. In relation to the journey of change, giving feedback to supervisors remained a challenge to most managers because often it was not valued:

*I am learning how to give constructive feedback to my seniors. I find it weird because they know exactly what's supposed to be done and most of the time you find that they don’t do. I wonder how I can give constructive feedback for my seniors. I become plastic, I switch off!* (Interview, hospital manager)

Furthermore, most managers felt that their supervisors would isolate and intimidate staff who openly expressed a varied opinion—for example, by being singled out for transfers to other facilities.

### Experiences and practicalities of implementing the intervention

#### Managers’ participation in and responses to the courses

In relation to the 1st level of Kirkpatrick’s model on reaction to the training, our observations, interviews and informal interactions revealed that participants found the training valuable. All eight participants were highly enthusiastic about the course, reporting that the concepts and tools shared were useful for their everyday work. The concepts most often mentioned included understanding health systems as complex systems, considering the software of the system, stakeholder analysis and engagement, reflective practice and the concept of achieving ‘small wins’ when initiating change.

The stakeholder analysis tool was highlighted as valuable in recognizing actors in the system and learning how to engage with them in understanding and implementing change. Managers also reported that the ‘Challenge Model’ was used in identifying strategic priorities to address identified challenges and develop work plans, providing them with a starting point for finding solutions specific to their contexts. Furthermore, the tools, taught concepts and work-based use of the Challenge Model enabled managers to develop strategies for drawing their teams into discussions, gaining the collective confidence to tackle what initially seemed overwhelmingly complex problems (Mansour *et al.*, 2010).

*This course is relevant because anything that is a game changer is relevant. There is some sort of toning down, there is some sort of humility that’s becoming more apparent. And, realizing that this is my bad point this is what I need to work on, that’s very important. Having insight of who you are compared to before thinking that you know it all that’s already a game changer. Then again adult learning every time there is that repetition you get a better result* (Interview, Hospital manager)

The communication and emotional skills training process was organized over several months, and therefore faced greater challenges in maintaining consistent attendance. Inconsistent participation over time was mainly attributed to being called away to other *ad hoc* meetings as opposed to disinterest in the course itself. Participants who were most consistent in attending and completing reflective assignments were female nurse managers, who were particularly passionate about their managerial roles and had a support network of close friends, colleagues or family members. These managers exhibited deep interest in the learning approach and talked about the need for persistence in putting the learning to practice.

#### Modes of learning: individual and collective learning

Perceived gains in knowledge, skills, attitude and confidence were in line with Kirkpatrick’s 2nd level of learning and encompassed both individual and collective learning as well as improved working relationships.

*when giving feedback I also ask people what they think, what are their opinions. Before I would sit down and tell them this is the word, this is the law, take it or leave it.* (Interview, Hospital Manager)

Participants talked about the transformative impact of the course in empowering managers with confidence and trust through: building listening skills; gaining better awareness of their own and colleagues’ emotions; empowering them to step back from automatic reactions when faced with emotional situations and consciously taking responsibility to communicate with awareness and empathy. According to these managers, such skills prepared them to handle conflicts better as they treated colleagues with greater respect and gave more constructive feedback.

### Skills and competencies acquired by managers

#### Building teams

According to the managers, the practical concepts from the trainings and experiences of being part of the learning site activities enabled them to start engaging others to buy into the vision and goals of their organisations. Examples included how, while prioritizing strategic areas for improvement in their organisations during the health systems training, the Challenge Model and the stakeholder analysis tool became useful resources for bringing team members together. The Model was also useful for team-based exercises providing peer learning space and more concerted effort towards shared goals.

Owing to renewed team norming, some managers described how they started acknowledging the value of others, tapping into shared conceptualisation of problems and solutions and collaborative decision-making. Relatedly, they shared examples of how delegating authority and responsibility to others in their organisation was a strategy for strengthening leadership.

*I was waiting for a progress report from a particular colleague whom I had assigned to do regarding an activity we had concluded. After several repeated inquiries the report was not ready and I expressed my concern to another senior colleague who made a remark that the said colleague was known to be difficult and a headache to work with.*

*Without emotional intelligence and self-awareness, I would have confronted the colleague in a harsh manner and demand for the report without any regard. I chose to engage with him through a one on one meeting, I noted the challenges and strengths different team members have, rather than side-lining him. I helped him in understanding what I expected out of him and importance of team work and role delegation* (Notes from a participant’s reflective diary)

*After devolution there was a direct connection between this office and the department heads. I will say the director, the chief and the Chief Nursing Officer, we could talk one to one. They would come on the ground, see what we are experiencing and I continued to lobby, they could make some spot checks and they appreciated that I had a problem or the hospital had a problem and from 33 nurses as we are speaking we are now 64, …that’s double* (Interview, sub-county nurse manager)

*One manager reported that she and the MCA (Member of County Assembly) have become friends. She attributed this to having a new group of MCAs who engaged in a different way and she often had opportunities to discuss development issues.* (Hospital manager, reflective meeting)

Managers also began engaging opinion leaders and draw on them as agents of change wherein change behaviour required collective action. This was often done by encouraging team-based decision-making, valuing inputs of team members and tapping into members’ readiness to accept responsibilities.

#### Improved relationship with colleagues and juniors

Recognizing others’ values and capacity led to improved relationships and a sense of solidarity in solving routine challenges by forging a united front with colleagues in tackling emerging problems. For instance, some nurse managers, recalling personal resilience during the nurses’ strike, explained how they used persuasion, incentives and social influence to appeal to, reassure and motivate some nurses to continue providing services to the most vulnerable. While recognizing the potential risks for nurses who continued to work during these difficult circumstances, there were no easy solutions to maintaining service provision.

Other managers recounted how they drew on their personal relationships with technical partners and other senior managers to enable resource mobilisation, which in turn ensured continued service provision. Managers strategically built allies with some of the political leaders involved in decision-making on health matters to get their buy-in.

*So actually, we had to strategize, also thankfully we also had relationships with our partners, because they are the key people holding our hands… to get work done. So, we’ve done a lot of strategizing, thinking…* (Interview, hospital manager)

These examples show how managers started recognizing how to use relationships to build support for their actions and appreciate how to manage different stakeholders at various levels of the system.

#### Role-modelling

Most facility managers narrated role-modelling positive behaviours learned from the courses as a way of exemplifying change agency and motivating others. For example, a hospital matron had to exercise skill in balancing and managing the relationships that influenced delivery of services during the strike period, by working long hours alongside the available staff:

*So being there for them (during the strike) doing what you can do, being with them, if its washing patients we do it together, role modeling.* (Interview, nurse-in-charge)

The perceived changes in behaviours and practices of health managers appear to have been facilitated by leveraging on existing collegiality, familiarity and friendships as an avenue to trade/ask for favours, share positive values and counsel.

*It is not about telling them that from now to we are going to do this, this and that but it starts by setting an example. With that they actually see you are actually very… you are an approachable person you are honest… even small things like you are always the first at work or something*. (Interview, hospital manager)

### Application and spread

To understand the value of the training beyond individuals, we explored organisational and team culture change linked to Kirkpatrick’s 3rd level of evaluation, specifically exploring scope in applying new thinking from the training.

More than half of the managers recounted experiences of spreading change, emphasizing growing confidence, e.g. educating others during Continuous Medical Education (CME), practicing ‘stepping back’, ‘using empathy’ and ‘giving constructive feedback’ both at work and at home. However, most reported needing more time to put those changes in place.

#### Problem-solving

Linked to improved confidence and increased agency, managers reported finding local solutions to routine problems rather than complaining about them or feeling helpless. In so doing they leveraged on relationships with external partners to get help with resources.

*We’ve done a lot of re-strategizing, thinking and…, because there are some days where, where we actually don’t have a vehicle, don’t have, small things like fuel for going to do our supervision. So, it’s just been getting the job done, talking to our partners, getting finances, getting vehicles …at least getting to our people….* (Interview, hospital manager)

But some of these efforts were undermined by how normalized practices undermined the need for new innovative practices:

*There are certain regulations for staff with disciplinary issues which involve transferring them [as described in our regulations and protocol], which often does not solve the problem but ends up transferring the problem elsewhere* (Diary entry, sub-county manager)

#### Sharing experiences

It was interesting to learn that most managers were using the learning from the course to adapt everyday routines, e.g. running routine problem-solving meetings using the listening and constructive feedback skills learned.

More enthusiastic managers reported acting as local champions in their organisations, sharing some of the learning during CME sessions with others who were not part of the original training:

*So, our aim was to give constructive feedback, we had sat down with the Sub County before like now then we come with those plans and then like we’re sitting with these people, we should learn the achievements the challenges and the groups give also contribution. Not me only [[M1: Yes]] the one who got the skills. But even them when all the time I sit with them in meetings I also teach them, like ooh here we went over this leadership we learnt this…stepping back, how to control emotions, the importance of apologising, so bringing up these issues every now and then.* (Interview, Sub-county manager)

#### Perceived sustainability for change

Recognizing that behavioural change is a long-term process, we sought to understand what would sustain change in line with Kirkpatrick’s levels 1–3 over time. We considered what the challenges of implementing new practices would be and sought managers’ recommendations of how to navigate such challenges.

To sustain learnt skills, managers reiterated the importance of senior management support, involvement in these training courses and in creating an organisational climate that supports emotional wellbeing for all workers. However, managers also noted that using the new skills to tackle complex problems contributed to job satisfaction, and deepened commitment to sustaining new practices.

*It’s not about what the county gives me but every time I find I can help someone find a solution to a problem that they have it makes me feel good. This has been my driving force* (Interview, sub-county health manager)

Despite positive strides, we noted underlying issues that threatened their ability to maintain application of learned skills. These included broken trust, especially between middle-level managers and senior managers, owing to unclear reporting and accountability hierarchies (following devolution) and the bureaucratic inertia to change at the frontlines. Other limiting factors were unstructured communication channels and constant political interference that threatened managers’ job security and undermined improvement efforts.

For the communications course, being together long enough to feel comfortable with others, and to be able to open up, was judged to be particularly crucial in enabling learning:

*For example, an MCA (Member of County Assembly) can demand for that patient to be referred…even when you think is not fit for referral. So, there is a lot of external pressure especially from the political class.* (Interview with Sub-county manager)

*But you see that one day or two days the bonding was so minimal for somebody to be very safe to let him or herself vulnerable to…in that environment. Maybe my recommendation is that, if the emotional intelligence module can be packed in one week so that people can be able to come up to bond, and actually express their feelings* (Interview, hospital manager)

Indeed concerns about the lack of support and poor communication prevented some managers from volunteering to participate in the course in the first place and contributed to sporadic attendance by others.

In summary, our evaluation shows that there were reports of increased confidence in work and communication methods, overall improvement in relationships with juniors and colleagues, improved skills in stopping automatic reactions, focusing on common goals and practical use of tools aimed at understanding the complexity of the system.

*You know, the one for communication… it has some series that one has to give their time, like you go then you come and there are things that you have to do to evaluate yourself. Others felt it was a bit of hard for them. Like me I wanted to take it, but I felt at times I may be needed [elsewhere], how many times will I have to do it? But it is a good course* (Interview, hospital administrator)

However, key obstacles to the implementation of the course, as well as sustained positive impact over the longer term, included pervasive hierarchical authority in the system and balancing competing priorities. While the training was designed to and could assist with some of these challenges, through, for instance, strengthening multidirectional communication and supporting identifying opportunities for organizational change, there are deeply rooted cultures of reluctance in questioning authority and questioning decisions over what ought to be prioritized in health systems We experienced such challenges in engaging the managers in developing these initiatives: authorisation and permission processes required for the attendance of training were often unclear, training activities were regularly interrupted by job demands and *adhoc* summons from senior managers, and there was an absence of broader organisational mechanisms to support emotional wellbeing. Given both the potential and challenges for initiatives like this in these contexts, these processes and impacts need longer term tracking, as we return to in the discussion.

## Discussion

Measuring the effectiveness of leadership development programmes and other initiatives aimed at improving functioning of health systems is challenging: systems are subject to a myriad of influences and constraints (Collins, 2001; Mcalearney, 2006), making it difficult to determine the chain of events linking to behavioural change(s), and to attribute elements of behavioural change(s) to particular interventions (Lemay and Ellis, 2008b). Nevertheless, given the potential importance of leadership development programmes for building everyday resilience in health systems, it is important that innovative context-sensitive initiatives are developed and evaluated. We sought to understand how an innovative leadership intervention, centred on strengthening knowledge and application of software leadership skills, assisted mid-level managers in the everyday problems they faced in conducting their roles in under-resourced health systems (Samra-Fredericks, 2003; Molyneux *et al.*, 2016).

Our analysis suggests that at an individual level, participating managers learned the practical skills of better communication including active listening, management of emotions and demonstrating respect and empathy. Individuals involved in the communication and emotional competence training considered these as ‘implementable’ social skills that enabled them to reconsider their automatic reactions and responses to emotional challenges, in turn strengthening their leadership and management. Specifically, development of these individual-level skills supported managers to better understand the emotions and automatic responses of team members, and encouraged greater open discussion and reflection with colleagues. This in turn appeared to help strengthen teams through supporting more positive relationships, creating organizational routines and processes that allowed collaborative decision-making and developing shared mindsets (Lengnick-Hall *et al.*, 2011). Managers also spread individual skills to team members and colleagues by role-modelling positive behaviour, delegating leadership and responsibilities to others and using the Challenge Model to create space for interaction and collective problem solving.

Our findings are in line with literature which recognizes that the complexity of managing relationships requires interventional approaches that strengthen leadership through combining face-to-face training with workplace-based activities, and that incorporate and build reflective practice processes individually and within teams (Millward and Bryan, 2005; Mccallin, 2003). Reflective practice in leadership programmes focused on the relational elements of leadership such as communication and team processes should improve understanding of the self, as well as the behaviours and relationships of the self in relation to others (Quinn, 2013; Cotter, 2014); something our training appears to have made progress towards.

The relevance and importance of leadership interventions as experienced by managers is suggested by our wider resilience work (Gilson *et al.*, 2017; Kagwanja *et al.*, 2020; Gilson *et al.*, 2020). Our overall conceptualisation of everyday resilience includes recognition of the system capacities needed to absorb, adapt and transform while responding to challenges, so that the system is strengthened through those responses. The three sets of capacities needed are: cognitive (the shared values, language and mindsets that enable collective problem-diagnosis and problem-solving); behavioural (the organizational routines and patterns that support response to challenges and actions to address them); and contextual (the network of interactions and resources that underpin response to challenges) (Lengnick-Hall *et al.*, 2011).

Through our intervention, which included a combination of individual- and team-level knowledge and skills-building activities as well as long-term engagement, a shared cognitive ability to reconceptualize problems emerged. Managers developed a sense of confidence and an ability to make decisions and act, which in turn supported teams to engage in collective problem-solving and action (Van Den Bossche *et al.*, 2011). This contributed to their ‘cognitive resilience capacity’ (Lengnick-Hall *et al.*, 2011). Moreover, the agency managers exercised in deploying routine and unconventional responses, drawing on learned resourcefulness and involving the unlearning of some habitual behaviours, are forms of ‘behavioural capacities’ integral to everyday resilience. For instance, managers’ role-modelling positive behaviour to team members represents Alvesson *et al*.’s characterisation of leadership as the ‘extraordinarisation’ of mundane activities, emphasizing how the exchanges between leaders and others in the system are vital to the functioning of teams and wider systems (Alvesson and Sveningsson, 2003; Uhl-Bien, 2011). The interventions—by leveraging on relationships and interactions with other actors within the system—were also cited as a useful strategy and appear to have also contributed to building contextual capacities. In this way, we can see that through impacting upon the daily micro-level practices of managers within the broader organisational, socio-political context in which they are embedded, the course contributed to collective capacities needed for everyday health system resilience and health system strengthening.

In previous learning sites work, we described a range of chronic and acute stressors facing the Kenyan health system including system-wide challenges (Kagwanja *et al.*, 2020) and organisational cultural constraints (Barasa *et al.*, 2017b). We described how mid-level managers developed relational strategies within existing social networks to cope with the crises associated with a rapidly devolving national health system (Nyikuri *et al.*, 2015; Kagwanja *et al.*, 2020). We suggest that leadership development initiatives such as the one we have described not only have the potential to strengthen leadership responses during particularly challenging times (such as crises related to devolution, strikes and Covid-19), but also to also begin to support the organisational cultural change(s) needed to sustain much-needed collective capacities for everyday system resilience.

Nevertheless, the success of leadership development initiatives such as ours in shifting collective behaviours and practices is threatened by the same challenging environments that the training can and should begin to transform. Although the value of developing the software dimensions of leadership cannot be overstated, we found that the practicality and sustainability of such skills was often threatened by unclear hierarchies, political interference and a pervasive culture of mistrust (Nyikuri *et al.*, 2017; Tsofa *et al.*, 2017). This threatened the translation of individual skills into collective organisational gains at team and system levels, as well as the sustainability of the gains. It is essential that those organisational cultures and contexts begin to be changed.

Our leadership interventions start to address this challenge by teaching managers how to use their power; encouraging managers to see that they have power, and that they cannot lead without knowing how and when to use that power (Espedal, 2017; Sarah, 2017). In so doing, we were trying to empower managers to see themselves as credible sources of authority and as change agents. Power relations in organisations define what leadership is and what it does by regulating the identities and roles of the incumbents (Alvesson *et al.*, 2008). Part of the process of gaining power and legitimacy is rooted in the ability to identify the emotions of oneself, others and of the situation, and modelling appropriate responses to influence others so as to legitimize actions taken. This is particularly crucial for mid-level managers who have to manage and balance their relations with senior managers and the juniors they lead (Nzinga *et al.*, 2018; Barasa *et al.*, 2016). Our course appeared to make some progress in empowering managers, albeit in a highly challenging context. This progress should continue to be tracked over time within ongoing learning site activities.

### Implications for policy and practice

**Figure 2.**
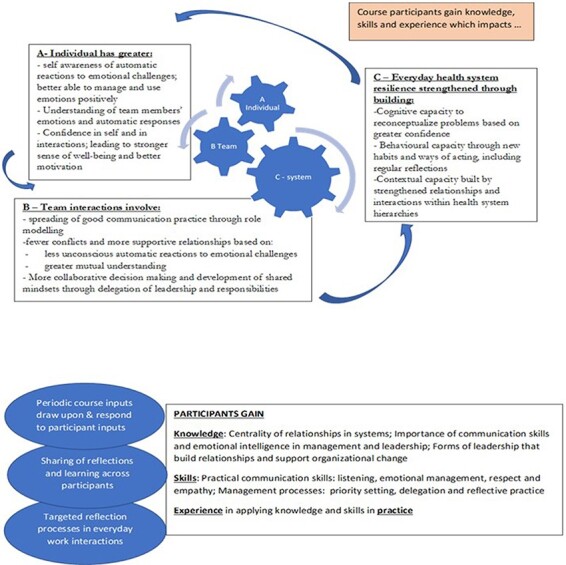
Theory of change depicting our conceptualisation of how the training influenced behaviour and practice

Our evaluation offers valuable insights for healthcare policy and practice in Kenya and in other LMIC contexts. First, leadership training ought to be focused on developing conceptual, behavioural and contextual capacities that can enable mangers to reframe routine challenges, and on building managers’ social and emotional skills in implementing change through teams and teamwork. Second, our work emphasizes the importance of work-based process training, creating dedicated safe spaces that encourage team reflection and sharing of lessons over time, while nurturing the soft skills that are essential in building constructive interpersonal relationships. Drawing from our experience, we propose a theory of change (Figure 2) to guide future intervention and evaluation.

Given both the potential and challenges for initiatives like this in hierarchical contexts, these processes and impacts of change also need longer term implementation and tracking. Recognizing some of the challenges we faced, time must be dedicated not only to teaching but also to periodic follow-up of participants in their workplaces, and recruiting senior managers as course participants and ultimately trainers. Any such initiatives and support processes must also be seen as part of a wider set of interventions aimed at strengthening relationships across systems, and ensuring that managers are supported with access to both the software and hardware essential to them in performing their roles.

In conclusion, this programme of work contributes to calls for better articulation of soft skills leadership training and highlights the benefits of personal and professional transformation from simply managing to influencing others (Cleary *et al.*, 2018; England, 2002). It is hoped that our embedded research approach to supporting health managers’ leadership capacities would be useful to practitioners and policymakers in building in contextualized leadership training and support in routine managerial processes. More importantly, this article supports the value of new forms of leadership that encourage teamwork and build relationships, enabling the emergence of the system capacities needed to adjust and develop agency in tackling routine problems to improve everyday health system resilience.

## Data Availability

The data underlying this article are available in the article.

## Endnotes

^1.^
During the study period there was a prolonged nation-wide nurses’ strike in Kenya, which led to reduced coverage of public health service provision and closure of primary healthcare facilities in the country for 5 mo. We have documented the nature and effect of this industrial strike elsewhere (Waithaka *et al.*, 2020). Prolonged health worker strikes in Kenya-perspectives and experiences of frontline health managers and local communities in Kilifi County. *International Journal for Equity in Health,* 19, 23.

## References

[R1] Alvesson M, Lee Ashcraft K, Thomas R. 2008. Identity matters: reflections on the construction of identity scholarship in organization studies. *Organization* 15: 5–28.

[R2] Alvesson M, Sveningsson S. 2003. Managers doing leadership: the extra-ordinarization of the mundane. *Human Relations* 56: 1435–59.

[R3] Alyahya MS, Norsiah B. 2013. Evaluation of effectiveness of training and development: the Kirkpatrick model. *Asian Journal of Business and Management Sciences* 2: 14–24.

[R4] Aragón AO. 2010. A case for surfacing theories of change for purposeful organisational capacity development. *IDS Bulletin* 41: 36–46.

[R5] Barasa E, Boga M, Kagwanja N et al. 2020. Learning sites for health system governance in Kenya and South Africa: reflecting on our experience. *Health Research Policy and Systems* 18: 44. 10.1186/s12961-020-00552-6 PMC7212564 32393340

[R6] Barasa EW, Cleary S, English M, Molyneux S. 2016. The influence of power and actor relations on priority setting and resource allocation practices at the hospital level in Kenya: a case study. *BMC Health Services Research* 16: 536. 10.1186/s12913-016-1796-5 PMC5045638 27716185

[R7] Barasa EW, Cloete K, Gilson L. 2017a. From bouncing back, to nurturing emergence: reframing the concept of resilience in health systems strengthening. *Health Policy and Planning* 32: iii91–iii94. 29149319 10.1093/heapol/czx118 PMC6626547

[R8] Barasa EW, Manyara AM, Molyneux S, Tsofa B. 2017b. Recentralization within decentralization: county hospital autonomy under devolution in Kenya. *PLoS One* 12: 1–18. 10.1371/journal.pone.0182440 PMC5542634 28771558

[R9] Chelagat T, Rice J, Onyango J, Kokwaro G. 2021. An assessment of impact of leadership training on health system performance in selected counties in Kenya. *Frontiers in Public Health* 8: 1–12. 10.3389/fpubh.2020.550796 PMC7956995 33732670

[R10] Cleary S, Toit AD, Scott V, Gilson L. 2018. Enabling relational leadership in primary healthcare settings: lessons from the DIALHS collaboration. *Health Policy and Planning* 33: ii65–ii74. 30053037 10.1093/heapol/czx135 PMC6037064

[R11] Collins DB. 2001. Organizational performance: the future focus of leadership development programs. *Journal of Leadership Studies* 7: 43–54.

[R12] Cotter RJ. 2014. Reflexive spaces of appearance: rethinking critical reflection in the workplace. *Human Resource Development International* 17: 459–74.

[R13] Cronin A, Alexander V, Fielding J, Moran-Ellis J, Thomas H. 2008. The analytic integration of qualitative data sources. In:Alasuutari P, Bickman L (eds). *The SAGE Handbook of Social Research Methods*. Finland: SAGE, 572–84.

[R14] Crowne KA, Young TM, Goldman B et al. 2017. Leading nurses: emotional intelligence and leadership development effectiveness. In: *Leadership in Health Services* 30: 217–32. 28693391 10.1108/LHS-12-2015-0055

[R15] Dart J, Davies R. 2003. A dialogical, story-based evaluation tool: the most significant change technique. *American Journal of Evaluation* 24: 137–55.

[R16] De Savigny D, Adam T. 2009. *Systems Thinking for Health Systems Strengthening*. Geneva, Switzerland: World Health Organization.

[R17] RESYST/DIAHLS learning site team. 2020. Learning sites for health system governance in Kenya and South Africa: reflecting on our experience. *Health Research Policy and Systems* 18: 1–12. 32393340 10.1186/s12961-020-00552-6 PMC7212564

[R18] Doherty J, Gilson L, Shung-King M. 2018. Achievements and challenges in developing health leadership in South Africa: the experience of the Oliver Tambo Fellowship Programme 2008–2014. *Health Policy and Planning* 33: ii50–ii64. 30053036 10.1093/heapol/czx155 PMC6037070

[R19] Edmonstone J. 2015. Developing healthcare leaders and managers: course-based or practice-based. *International Journal of Healthcare* 1: 9.

[R20] Egger D, Ollier E. 2007. Managing the Health Millennium Development Goals–the challenge of management strengthening: lessons from three countries: report on an international consultation on strengthening health leadership and management in low-income countries, Accra, Ghana. Making Health Systems Work, World Health Organization.

[R21] Elloker S, Olckers P, Gilson L, Lehmann U. 2012. Crises, routines and innovations: the complexities and possibilities of sub-district management: leadership and governance. *South African Health Review* 2012: 161–73.

[R22] England D, 2002. Inner leadership–personal transformation. *Industrial and Commercial Training* 34: 21–7.

[R23] Espedal B. 2017. Understanding how balancing autonomy and power might occur in leading organizational change. *European Management Journal* 35: 155–63.

[R24] Fulop L, Mark A. 2013. Relational leadership, decision-making and the messiness of context in healthcare. *Leadership* 9: 254–77.

[R25] Gilson L, Agyepong IA. 2018. Strengthening health system leadership for better governance: what does it take? *Health Policy and Planning* 33: ii1–ii4. 30053034 10.1093/heapol/czy052 PMC6037056

[R26] Gilson L, Barasa E, Nxumalo N et al. 2017. Everyday resilience in district health systems: emerging insights from the front lines in Kenya and South Africa. *BMJ Global Health* 2: e000224. 10.1136/bmjgh-2016-000224 PMC5656138 29081995

[R27] Gilson L, Ellokor S, Lehmann U, Brady L. 2020. Organizational change and everyday health system resilience: lessons from Cape Town, South Africa. *Social Science and Medicine* 266: 113407. 10.1016/j.socscimed.2020.113407 PMC7538378 33068870

[R28] Kagwanja N, Waithaka D, Nzinga J et al. 2020. Shocks, stress and everyday health system resilience: experiences from the Kenyan coast. *Health Policy and Planning* 35: 522–35. 32101609 10.1093/heapol/czaa002 PMC7225571

[R29] Kebede S, Mantopoulos J, Ramanadhan S et al. 2012. Educating leaders in hospital management: a pre-post study in Ethiopian hospitals. *Global Public Health* 7: 164–74. 21259143 10.1080/17441692.2010.542171

[R30] Kerr R, Garvin J, Heaton N, Boyle E. 2006. Emotional intelligence and leadership effectiveness. *Leadership and Organization Development Journal* 27: 265–79.

[R31] Kirkpatrick D 1994. *Evaluating Training Programs: The Four Levels*. San Francisco, CA: Berrett-Koehler.

[R32] La Duke P. 2017. How to evaluate training: using the Kirkpatrick model. *Professional Safety* 62: 20.

[R33] Lemay N, Ellis A. 2008a. Leadership can be learned, but how is it measured. *Management Sciences for Health* 8: 1–29.

[R34] Lemay N, Ellis A. 2008b. Leadership can be learned, but how is it measured? *Management Sciences for Health Occasional Papers* 8: 1–29.

[R35] Lengnick-Hall CA, Beck TE, Lengnick-Hall ML. 2011. Developing a capacity for organizational resilience through strategic human resource management. *Human Resource Management Review* 21: 243–55.

[R36] Loughran JJ. 2002. Effective reflective practice: in search of meaning in learning about teaching. *Journal of Teacher Education* 53: 33–43.

[R37] Mansour M, Mansour JB, Swesy AHE. 2010. Scaling up proven public health interventions through a locally owned and sustained leadership development programme in rural Upper Egypt. *Human Resources for Health* 8: 1. 10.1186/1478-4491-8-1 PMC2822741 20205749

[R38] Mcalearney AS. 2006. Leadership development in healthcare: a qualitative study. *Journal of Organizational Behavior: The International Journal of Industrial, Occupational and Organizational Psychology and Behavior* 27: 967–82.

[R39] Mccallin A. 2003. Interdisciplinary team leadership: a revisionist approach for an old problem? *Journal of Nursing Management* 11: 364–70. 14641717 10.1046/j.1365-2834.2003.00425.x

[R40] Millward LJ, Bryan K. 2005. Clinical leadership in health care: a position statement. *Leadership in Health Services* 18: 13–25. 10.1108/13660750510594855 15974507

[R41] Molyneux S, Tsofa B, Barasa E et al. 2016. Research involving health providers and managers: ethical issues faced by researchers conducting diverse health policy and systems research in Kenya. *Developing World Bioethics* 16: 168–77. 27699954 10.1111/dewb.12130 PMC5298022

[R42] MSH, U., Mom Services and Moph Sanitation. 2008. Report on management and leadership development gaps for Kenya health managers.

[R43] Nyikuri M, Tsofa B, Barasa E, Okoth P, Molyneux S. 2015. Crises and resilience at the frontline—public health facility managers under devolution in a sub-county on the Kenyan Coast. *PLoS One* 10: e0144768. 10.1371/journal.pone.0144768 PMC4687913 26696096

[R44] Nyikuri MM, Tsofa B, Okoth P, Barasa EW, Molyneux S. 2017. “We are toothless and hanging, but optimistic”: sub county managers’ experiences of rapid devolution in coastal Kenya. *International Journal for Equity in Health* 16: 113. 10.1186/s12939-017-0607-x PMC5599878 28911332

[R45] Nzinga J, Mbaabu L, English M. 2013. Service delivery in Kenyan district hospitals–what can we learn from literature on mid-level managers? *Human Resources for Health* 11: 10. 10.1186/1478-4491-11-10 PMC3599555 23442524

[R46] Nzinga J, Mcgivern G, English M. 2018. Examining clinical leadership in Kenyan public hospitals through the distributed leadership lens. *Health Policy and Planning* 33: ii27–ii34. 30053035 10.1093/heapol/czx167 PMC6037084

[R47] Ortiz A. 2010. A case for surfacing theories of change for purposeful organisational capacity development. *IDS Bulletin* 41: 36–46.

[R48] Quinn B. 2013. Reflexivity and education for public managers. *Teaching Public Administration* 31: 6–17.

[R49] Reich MR, Javadi D, Ghaffar A. 2016. Introduction to the special issue on “effective leadership for health systems”. *Health Systems and Reform* 2: 171–5. 31514592 10.1080/23288604.2016.1223978

[R50] Samra-Fredericks D. 2003. A proposal for developing a critical pedagogy in management from researching organizational members’ everyday practice. *Management Learning* 34: 291–312.

[R51] Sarah S. 2017. *Moderating Effect of Organizational Culture on the Relationship between Organizational Justice, Job Autonomy and Organizational Cynicism*. Universiti Utara Malaysia. Unpublished thesis, accessed through https://core.ac.uk/download/pdf/268144034.pdf

[R52] Sheikh K, Gilson L, Agyepong IA et al. 2011. Building the field of health policy and systems research: framing the questions. *PLoS Medicine* 8: 1–5. 10.1371/journal.pmed.1001073 PMC3156683 21857809

[R53] Tsofa B, Goodman C, Gilson L, Molyneux S. 2017. Devolution and its effects on health workforce and commodities management–early implementation experiences in Kilifi County, Kenya. *International Journal for Equity in Health* 16: 169. 10.1186/s12939-017-0663-2 PMC5599882 28911328

[R54] Uhl-Bien M. 2011. Relational leadership theory: exploring the social processes of leadership and organizing. In:Werhane P, Painter-Morland M (eds). *Leadership, Gender, and Organization*. Netherlands: Springer, 75–108.

[R55] Van Den Bossche P, Gijselaers W, Segers M, Woltjer G, Kirschner P. 2011. Team learning: building shared mental models. *Instructional Science* 39: 283–301.

[R56] Waithaka D, Kagwanja N, Nzinga J et al. 2020. Prolonged health worker strikes in Kenya-perspectives and experiences of frontline health managers and local communities in Kilifi County. *International Journal for Equity in Health* 19: 23. 10.1186/s12939-020-1131-y PMC7011250 32041624

[R57] Waweru E, Opwora A, Toda M et al. 2013. Are health facility management committees in Kenya ready to implement financial management tasks: findings from a nationally representative survey. *BMC Health Services Research* 13: 1–14. 24107094 10.1186/1472-6963-13-404 PMC3853226

